# Protecting exons from deleterious R-loops: a potential advantage of having introns

**DOI:** 10.1186/1745-6150-2-11

**Published:** 2007-04-25

**Authors:** Deng-Ke Niu

**Affiliations:** 1 Ministry of Education Key Laboratory for Biodiversity Science and Ecological Engineering, College of Life Sciences, Beijing Normal University, Beijing 100875, China

## Abstract

**Background:**

Accumulating evidence indicates that the nascent RNA can invade and pair with one strand of DNA, forming an R-loop structure that threatens the stability of the genome. In addition, the cost and benefit of introns are still in debate.

**Results:**

At least three factors are likely required for the R-loop formation: 1) sequence complementarity between the nascent RNA and the target DNA, 2) spatial juxtaposition between the nascent RNA and the template DNA, and 3) accessibility of the template DNA and the nascent RNA. The removal of introns from pre-mRNA reduces the complementarity between RNA and the template DNA and avoids the spatial juxtaposition between the nascent RNA and the template DNA. In addition, the secondary structures of group I and group II introns may act as spatial obstacles for the formation of R-loops between nearby exons and the genomic DNA.

**Conclusion:**

Organisms may benefit from introns by avoiding deleterious R-loops. The potential contribution of this benefit in driving intron evolution is discussed. I propose that additional RNA polymerases may inhibit R-loop formation between preceding nascent RNA and the template DNA. This idea leads to a testable prediction: intermittently transcribed genes and genes with frequently prolonged transcription should have higher intron density.

**Reviewers:**

This article was reviewed by Dr. Eugene V. Koonin, Dr. Alexei Fedorov (nominated by Dr. Laura F Landweber), and Dr. Scott W. Roy (nominated by Dr. Arcady Mushegian).

## Background

### A brief introduction on the potential cost and benefit of introns

Introns are intervening sequences that are spliced out of RNA transcripts. Four major classes of introns are recognized: group I introns, group II introns, tRNA/archaeal introns, and spliceosomal introns. Introns are found in all major groups of organisms on earth from bacteriophages to mammals [1], and reach densities of several introns per gene in a variety of eukaryotic lineages [2]. However, no general functional or evolutionary role for introns has been well established. Introns may represent nearly neutral 'junk' DNA [3], however they presumably carry at least some selective cost owing to extra energy and time expenditure during replication and transcription [4,5].

The large number of introns in eukaryotic genomes hints that they may confer some selective advantages to overweigh their costs [6]. Various potential selective advantages that might be conferred by introns have been previously proposed: facilitating exon shuffling in the origin and evolution of proteins, providing the possibility of generating alternatively spliced coding messages, increasing the rate of recombination, harboring regulatory elements, acting as signals for nonsense-mediated decay and mRNA transport from the nucleus, and distinguishing functional mRNA from arbitrary RNA transcript, etc [2,6-14]. Recently, it is proposed that fortuitous intron invasions following the origin of mitochondria may bring on a strong selective pressure for the origin of various eukaryotic features including the nucleus, the spliceosome, linear chromosomes, telomerase, and the ubiquitin signaling system [15-17]. Here I propose another potential common benefit to introns: maintaining genome stability by avoiding deleterious R-loops formed during transcription.

### Deleterious R-loops and potential mechanisms to avoid them

**Figure 1:**
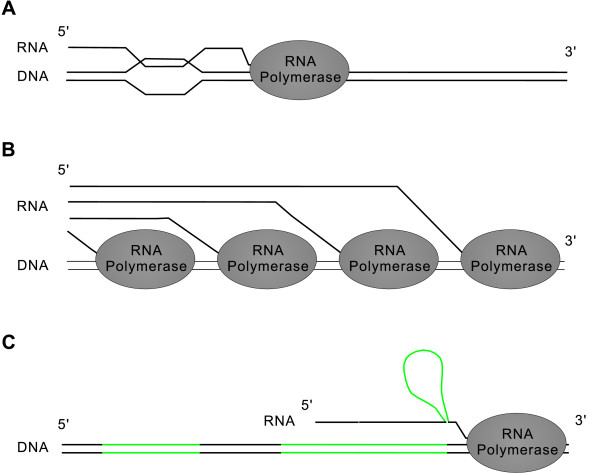
Schematic views of a transcriptional R-loop and two potential mechanisms to avoid it. (A) Nascent RNA re-anneals with the template DNA strand forming an R-loop. (B) A crowded DNA is difficult in forming R-loops with the nascent RNA molecules. (C) The effects of introns in avoiding R-loops. The green lines represent introns while the black lines represent the exons. The first intron of the nascent RNA has been spliced out while the second intron is being spliced. Removal of introns reduces the complementarity of the RNA molecule with the template DNA, meanwhile tethering exon together during transcription prevent the spatial juxtaposition between a nascent RNA and the template DNA.

The R-loop is a structure in which RNA invades and pairs with one strand of DNA to form an RNA-DNA hybrid (Fig. 1A) [18-20]. During transcription, the nascent RNA has the inherent capacity to form an R-loop with the template DNA strand [18-20]. In the *in vitro *transcription of some sequences, 42%–63% of the template DNA molecules form R-loops with nascent RNAs [20]. Recent evidence suggests that the transcriptional R-loops cause DNA strand breaks, rearrangements, and other types of DNA damage such as deamination [19,21,22]. Along with DNA topology [18], I expect that at least three factors are potentially required for the formation of an R-loop: (i) sequence complementarity between the nascent RNA and the target (template) DNA; (ii) spatial juxtaposition between the nascent RNA and the template DNA; (iii) accessibility of both the nascent RNA and the DNA template (i.e. both must not be paired or covered). Mainly based on the third factor, several potential mechanisms were previously proposed to inhibit R-loop formation [19,23]. Formation of stable stem-loop within nascent RNA may competitively inhibit hybridization between the RNA molecule and its DNA template. tRNA and rRNA genes may be protected from R-loops in this way. In addition, the nascent RNA can be separated from its DNA template by various proteins or protein complexes. In bacteria, translation is closely coupled to transcription, so the nascent mRNA is presumably often insulated by trailing ribosomes. In the absence of a translating ribosome, Rho factor can bind the nascent mRNA, disturbing R-loop formation. In eukaryotes, transcription and translation are decoupled. TREX (transcription/export) complex attached to the transcript during transcription in yeasts and serine-arginine-rich (SR) proteins recruited during splicing in animals have been shown to separate nascent mRNAs from their templates [21,22,24,25]. In this paper, I propose that RNA polymerases and introns may represent two additional potential important mechanisms to inhibit R-loop formation.

## Presentation of the hypothesis

### RNA polymerases and R-loop avoidance

As R-loop formation is a transcription-related phenomenon, is highly expressed genes more liable to form R-loops with their transcripts? In transcription bubble, nascent RNA is paired with the DNA template. But such short DNA:RNA hybrids are unlikely the cause of transcriptional R-loops. Some evidence has shown that nascent RNA molecules are separated from the template DNA by RNA polymerase after it has emerged from the exit channel of the RNA polymerase [26,27]. Thus the transcriptional R-loops should be generated by re-annealing of the nascent transcript with the upstream region of the DNA template (Fig. 1A). If the DNA template is crowded with trailing RNA polymerases, nascent RNA molecules will have difficulty in binding template DNA, disrupting R-loop formation (Fig. 1B). The crowded RNA polymerases on DNA template is not just a speculation. In exponentially growing cells, the RNA polymerases are very closely spaced. An extreme case was reported as 165 polymerases on a 6.74 Kb rRNA gene, i.e. one polymerase every 41 nt [28]. As the footprint of elongating RNA polymerases is about 35 nt [29], there are very few nucleotide residues uncovered in busily transcribed genes. The size of R-loops, as shown by electron microscopy, ranges from 150 bp to 500 bp [20]. So the busily transcribed genes should be protected from R-loops by RNA polymerase. It seems that intermittently transcribed genes and genes with stalled transcription are more liable to be damaged by R-loops.

The transcription processes in starving cells are likely to be prolonged because of substrate- or energy-limitation. According to the above hypothesis, the genes being transcribed in starving cells are liable to be damaged by transcriptional R-loops. In facts, there are many observations dating back to 1988 showing starved cells experience much (tens or even hundreds of times) higher mutation rates than fast-growing cells [30-34]. Consistent with increased R-loop formation contributing to this elevated mutation rate, much evidence suggests that the mechanisms of starvation-induced damages and transcriptional R-loop caused damages are similar: both processes involve recombination and DNA double-strand breaks [18,19,21,22,25,35-37].

### Avoid transcriptional R-loops by introns

The rate of ectopic recombination between DNA molecules declines as the homology length decreases [38]. Similarly, the efficiency of the hybridization between RNA molecules and its DNA template depends on the length of complementary sequences. The removal of introns is apparently an efficient way to reduce the complementarity between nascent RNA and the template DNA without changing the coded genetic information, and thus an efficient way to inhibit R-loop formation. Particularly in mammalian genomes where the coding exons are present as small islands in a sea of noncoding introns, the complementarity between nascent RNA and the template DNA is exceedingly reduced by removal of introns. It can be conjectured that small exons are favored in avoiding deleterious R-loops. Consistently, long exons are more prone to the transcriptional defects [39] that have been shown to be caused by R-loops [21]. Although large exons can be found throughout multicellular and unicellular eukaryotes, they are only a small proportion of the genes in each genome [40]. On the other side, long introns would protect the flanking exons more efficiently than small introns. Long introns in highly or quickly expressed genes are not favored in the selection of minimizing the energetic and time costs of gene expression [4,5,41,42]. But in weakly/slowly expressed genes, the selection for economy should be very weak. So the relatively longer introns in weakly/slowly expressed genes may be partially attributed to R-loop avoidance [4,5].

Similar ideas were previously published by other researchers. The fragmentation of a gene into exons may protect the coding sequence from recombination with its own processed pseudogenes [13,14]. Fedorov and Fedorova [10] proposed that, in the ancient RNA world, the cells may benefit from introns by differentiating translating RNA molecules from the corresponding inheritable RNA.

Recent work has revealed that intron splicing usually occurs coincident with transcription, beginning just after transcription of the sequence to be spliced ([43], with some exceptions [44]). Under this model, splicing would act to quickly reduce the complementarity between the nascent RNA and the template DNA. Meanwhile, splicing would quickly move the transcribed sequence away from the corresponding segment of template DNA, effectively avoiding R-loop formation.

Removal of introns from pre-mRNAs that are still undergoing transcription makes the pre-mRNA much shorter than the corresponding DNA, avoiding spatial juxtaposition between the nascent RNA and the template DNA. The pre-mRNA except the last synthesized exon is pulled 3'-side away from the corresponding genomic DNA regions (Fig. 1C). Recent studies show that the pre-mRNA exons are held together during transcription [45-47]. Thus, even if intron splicing is slowed down for some reason (for instance due to weak splicing signals), the exons could still be pulled 3'-side away from the corresponding genomic DNA regions (Fig. 1C). Certainly, the DNA and the nascent RNA are not rigid; they may be bent or flexed. Although I am not sure whether it is enough to inhibit the formation of R-loops, at least, the pull-mRNA-away can disturb R-loop formation.

Group I and group II introns have stable secondary structures [1,48,49]. The 5'-side exons of a group I/II-intron-containing pre-mRNA are also pulled 3'-side away from the genomic DNA, similar to tethering exons together by transcription complex [45-47]. More importantly, the spatial structures of group I/II introns may act as spatial obstacles for the formation of R-loops between nearby exons and the genomic DNA (the spatial structure of group I intron is shown in reference [50]).

The inherent stem-loop secondary structures of rRNAs are likely to inhibit the formation of R-loops [23]. As the stability of double helix comes partially from base stacking, I am not sure whether the short stem-loop secondary structures of tRNA molecules are more stable than continuous RNA:DNA double strand. The effects of R-loop avoidance by short stem-loop structures (like those in tRNA molecules) is doubtful [23]. But the long stem-loop structures of rRNAs are likely to play such role. In mRNAs, formation of such long stable structures is inhibited due to their translation: first, because coding meaning constrains the DNA sequence; secondly, because stable stem-loop structures may stall the translating ribosome, and trigger mRNA degradation [51,52]. Interestingly, the intron retained in cytoplasmic *HAC1 *mRNA has a stable stem-loop [44]. As such, the risk of R-loop formation between *HAC1 *mRNA and its template DNA may be reduced by the presence of the intron even if the intron is not removed immediately after transcription.

## Implications for intron evolution

As transcription and translation are coupled in archaebacteria as that in bacteria [53], nascent mRNAs in an archaebacterial cell may also be insulated by trailing ribosomes. Therefore, no matter the nuclei of eukaryotes was originated from bacterial genome or archaebacterial genomes, the origin of nucleus decoupled transcription and translation and so would require new mechanisms to avoid R-loop formation. The possible importance of R-loop avoidance to intron evolution in early eukaryotes depends on the scenarios of nucleus origin and the abundance of introns in early eukaryotic genome.

While spliceosomal intron origin remains debated, accumulating evidence suggests that the spliceosomal introns in eukaryotic nuclear genomes descended from group II introns [15,16,48,54]. If the origin of nucleus was triggered by invasion of group II introns after the endosymbiosis of mitochondria [15-17], the spliceosome and SR proteins evolved after the origin of nuclear introns. At the stage when transcription and translation were decoupled but the splicing factor SR proteins had not evolved, introns may be the only mechanism to prevent R-loop formation. The initial invasion of group II introns (i.e. before the origin of nucleus) should be under purifying selection [55] (see the comments of A.M. Poole for reference [16]), but intron expansion after the origin of nucleus would be favored by natural selection to maintain the genome stability. The alternative scenario is that the origin of nucleus was driven by other evolutionary pressures, selective advantages, or even before the symbiosis of mitochondria [17,56,57]. Transcription and translation were decoupled before the invasion of group II introns. New mechanisms were thus required to prevent deleterious R-loops. Both intron invasion (of group II introns from mitochondrial ones or by horizontal gene transfer from prokaryotes) and intron expansion would be favored by natural selection.

In both scenarios, there should be a strong selective force for intron expansion at the early stage of eukaryotic evolution. Once other mechanisms like SR proteins evolved to prevent transcriptional R-loops, the selective force for intron gain or against intron loss would be weakened. This speculation is consistent with the current consensus that the introns proliferate in early eukaryotic evolution while intron loss occurred predominantly in subsequent evolution [2,16,58-65].

Certainly, there is still the possibility that spliceosomal introns have existed since or even before the origin of cells, and were lost from prokaryotes because of strong selection for rapid reproduction [66]. If so, I suspect that the loss of introns from prokaryotic genes should be accompanied by the evolution of an efficient way to avoid R-loop formation, e.g. coupling transcription and translation.

The TREX complex used by yeast *Saccharomyces cerevisiae *to avoid R-loop formation is recruited onto mRNA during transcription [19,16]. Is it possible that the early eukaryotic ancestor used the TREX to keep mRNA away from the corresponding DNA? As the eukaryotic ancestor seems to be rich in intron [2,16,61-64], it is more likely that TREX replaced the SR proteins as a result of enormous intron losses in evolution.

According to this hypothesis, introns may be selectively maintained in evolution even if their sequences are not conserved. Despite the existence of the energetic and time costs [4,5,41,42], a minimal length of introns [67] must be maintained. It can be predicted that during compacting genomes in the evolution of some microorganisms, reducing intron size should be more prominent than reducing intron number. This is exemplified by the chlorarachniophyte nucleomorph, which has essentially the same intron density as free living green plants, but dramatically reduced intron size [68,69]. Another prediction is that the intermittently transcribed genes and genes with frequently prolonged transcription should have higher intron density (intron-number/mRNA-length) than other genes in the same genome. But the intermittently transcribed genes and genes with frequently prolonged transcription should be cautiously defined in further studies.

If introns can prevent transcription-associated genomic instability, the intronless genes are expected to be more risky than intron-containing genes. A compensating mechanism is to separate the mRNA more efficiently by proteins recruited during transcription and/or pre-mRNA processing. In fact, the intronless mRNAs have a significantly higher frequency of SR protein binding sites [70]. Similarly, I suspect that the extraordinarily large exons [40] are also rich in such binding sites.

Dr. Scott Roy thought more deeply on this subject while reviewing this paper. In his review (attached after the main body of this paper), readers can find comparisons of this hypothesis with previously ones, and a quantitative estimation for the benefit of R-loop avoidance.

## Conclusion

The major groups of introns, Group I/II introns and spliceosomal introns, may have the effect of protecting exons from deleterious R-loops. Although speculative and somewhat naive, I propose that the benefit may be selected as a function of introns in evolution. It is also possible that avoiding R-loops by the presence of introns is just a subsequent and secondary property, which came in well after introns and splicing machinery became established. Till now, I am not sure how strong the effect of avoiding R-loops is, and how much the benefit has driven the evolution of introns. Regardless of the quantitative uncertainty, this is the first time to propose that introns may have the effect of protecting exons.

## Competing interests

The author(s) declare that they have no competing interests.

## Reviewers' comments

### Reviewer's report 1

Eugene V. Koonin, National Center for Biotechnology Information, National Library of Medicine, National Institutes of Health, Bethesda, MD 20894, USA

We do not know why all eukaryotes (so far) have introns; what seems, more or less, certain, is that there is a complex web of neutral and selective factors underlying this quintessential feature of eukaryotes. So any reasonable proposal on the raison d'etre of introns is of interest. The hypothesis discussed in this paper, namely, that introns prevent the formation of deleterious R-loops by limiting, via cotranscriptional splicing, the amount of nascent RNA that is available for hybridization with the genomic DNA at any given time, is one such idea, and welcome in that capacity. However, I cannot help thinking that the idea is rather weak. Indeed, introns seem like an awfully expensive way to avoid R-loop formation. Why not simply sequester the growing RNA chain via the polyadenylation complex and the nucelocytoplasmic export machinery? In fact, eukaryotes do just that. Furthermore, there are many virtually intronless eukaryotes (although no literally intronless ones) in which introns cannot protect genomes from R-loops but which nevertheless survive just fine. Again, to the extent R-loops are, indeed, a menace, they are avoided by sequestering the nascent transcripts in a variety of complexes. One could argue, with rather good reasons, that these sequestering mechanisms themselves descend from the ancestral splicing machinery, so the role of introns in the avoidance of R-loop formation might have been greater at the early stages of eukaryotic evolution. I believe this is what the author implies toward the end of the paper. Nevertheless, at this stage, I cannot avoid the conclusion that the proposed mechanism, if real, only can be a minor contributor to the evolution of eukaryotic gene structure. I find it commendable that, in the concluding remarks, the author is very candid about the uncertainty with respect to the actual importance of R-loop avoidance.

*Author response: I agree with the comments. The actual importance of R-loop avoidance by introns is uncertain now. Further studies are required for a conclusion*.

### Reviewer's report 2

Alexei Fedorov, Director of Bioinformatics Lab, the University of Toledo, Toledo, OH 43614-5809, USA (nominated by Dr. Laura F Landweber)

This paper describes one of the most intriguing and incomprehensible questions in molecular biology – origin and evolution of introns. The author shows deep understanding of multiple problems associated with existence of exon/intron gene structures. After 25-years of intron early-or-late debate it is absolutely clear that nobody can prove or disprove a particular intron evolution hypothesis among a number of proposed ones. Thus, I do not expect a paper to resolve this very intricate problem and welcome any new fresh look on this subject.

I read this MS with interest and think that it deserves publication. However, I am disappointed about the absence of any quantitative estimations of the effect of hybridization of transcripts with their DNA matrixes. Even in the conclusion the author writes: "I am not sure how strong the effect of avoiding R-loops is, and how much the benefit has driven the evolution of introns". This is the weakest side of the MS. The author should try to provide as much quantitative estimation as possible. For example, on page 4, in the last paragraph of the Background section, the author writes: "...there are many observations since 1988 that starved cells experience high frequency of mutations." Is it 5–10% or 100–200% increase? This and all similar places must have numerical estimations which would significantly increase the value of the paper and the hypothesis. For another example on the same issue – see page 7 (Section: "Avoid transcriptional deleterious R-loops by introns", last paragraph), the statement: "At least the translated regions of most mature mRNAs are unlikely to have stable secondary structures". This statement also lacks any quantification. However, if the author takes modern RNA folding software package (M-fold, S-fold, for instance) and studies local 2D structures in exons vs. introns; it appears that many exons have energetically stable secondary structures comparable to those inside introns. After examination of thousands of exonic and intronic sequences, I can claim that there is only a subpopulation of exons (about 25–30% of the entire human pool) that do not exhibit strong secondary folding (< -20 kcal/mol per 100 bp). The rest of human exons are comparable to introns on this property (our yet unpublished results).

*Author response: Quantitative estimations are expected by any hypothesis advocator. In present case, previous experimental studies provided very little quantitative information. Also limited by my academic capacity, I am not able to do quantitative estimation. Fortunately, Dr. Roy approached a quantitative estimation in his review of this paper. His estimation is a very helpful supplement of my manuscript. I revised this manuscript with more numerical descriptions of previous experimental results*.

*On the stable secondary structures of RNAs, there is another uncertainty. All that we know was proposed by Gowrishankar and Harinarayanan in their paper (Mol Microbiol 2004, 54:598–603), but not demonstrated. As the stability of double helix comes partially from base stacking, the short stem-loop secondary structures of tRNAs seem less stable than continuous RNA:DNA double strand. I doubt the importance of R-loop avoidance by short stem-loop structures (like those in tRNA molecules). So I revised the statement*.

Finally, I agree with the author that introns could help in prevention of hybridization of transcripts with their original matrixes. In fact, we published a similar hypothesis but for RNA world (JME 2004, 59:718–721).

*Author response: I was unaware of that paper. Now I realize that it has similar ideas, and so I cite it in the body of this hypothesis. Meanwhile, I add several other related references*.

### Reviewer's report 3

Scott W. Roy, Allan Wilson Centre for Molecular Ecology and Evolution, Massey University, Palmerston North, New Zealand (nominated by Dr. Arcady Mushegian).

I have no idea whether Dr. Niu's hypothesis is true, but it is certainly intriguing and deserves to be widely read.

To me, a (or perhaps the) central mystery of intron evolution concerns the unique apparent proliferation (as well as transformation) of type II introns in early eukaryotes, with no similar event in any prokaryotic lineage nor perhaps in subsequent eukaryotic evolution. Dr. Niu's hypothesis offers a possible solution to this quandary: intron proliferation would have ameliorated the mutation rate increase associated with separation of transcription and translation brought on by the nucleus.

This hypothesis is important in that it is formally different from many previous hypotheses in that it (i) invokes positive selection to explain intron spread, and (ii) proposes that this positive selection solves a problem that would be unique to (early) eukaryotes.

The hypothesis is different from many previous attempts to explain intron proliferation within early eukaryotes due either to (i) increased mutation rates (for instance due to ongoing leakage of endosymbiont DNA into the pre-nucleus in the model of Martin and Koonin; due to increased TE proliferation due to sexual reproduction in the model of Hickey, Poole, and in unpublished ideas by myself); (ii) decreased population size (as put forward by Lynch and Richardson as well as by Martin and Koonin); or (iii) decreased selection against introns (if for instance eukaryotic ancestors tended more to be K-strategists than prokaryotes, or due to increased intergenic regions (though this again begs the question of where these intergenic regions came from if not from transposable element spread itself)).

At the same time, the hypothesis is different from many other previous ideas that see an advantage for introns, in that it proposes an advantage that would have been (i) immediate, rather than long-term; and (ii) would have been unique to early (or pre) eukaryotic ancestors. Many previous ideas for an advantage for introns (exon shuffling, allowing for alternative splicing, harboring regulatory elements) generally rely on subsequent additional mutations (for instance an actual exon shuffling event) which are expected to occur at low rates and therefore are unlikely to have led to the initial fixation of the intron itself. Other ideas have proposed types of positive selection are not specific to early eukaryotes (Forsdyke's ideas, ideas about distinguishing coding RNA from mRNA in the RNP world, distinguishing mRNA from other RNA, etc.). Other hypotheses such as Lynch and colleagues' ideas about intron spread being facilitated by NMD invoke eukaryotic-specific processes (NMD), however these processes themselves are likely largely required by introns' presence (i.e. intron presence likely leads to a higher rate of production of aberrant transcripts, thus initial intron spread seems more likely to explain NMD than the other way around).

By contrast, Niu's idea suggests a reason for general positive selection for intron spread that is specific to early eukaryotes. Given the ubiquity of introns in eukaryotes, the dearth of hypotheses based on positive selection is striking, and therefore any such hypothesis is important and at the very least thought-provoking.

Now, to the hypothesis itself. Among the host of possible objections to the hypothesis that I can imagine, I believe that fairly satisfying answers are possible.

The first is overkill: faced with the seemingly simple challenge of segregating nascent transcripts from DNA, why would evolution have devised as elaborate and seemingly problematic a mechanism as the spliceosomal system, rather than a simpler and presumably more efficient TREX-like transcript-coating mechanism? However, type II introns were likely available in the early eukaryotic nucleus (likely imported with the mitochondrion); type II intron transpositions that were overall favored would fix, intron numbers (and thus genome-wide transposition rates) would increase, and introns would saturate the genes. The shift towards trans-splicing would then only come secondarily. Given the positive-feedback dynamics of intron proliferation, it could be quite rapid, conceivably requiring less time than emergence of an RNA-coating protein (complex) which would need to distinguish mRNAs from non-coding functional RNAs in the cell. Introns then could emerge as the first line of defense, with TREX-like coating mechanisms only later taking over the role of transcript protection in some lineages.

The second concern is whether the selective advantage proposed, of reducing the mutation rate in coding sequence upstream of the intron site, is likely to be sufficiently strong to overcome drift. In general, selection will be efficient if the selective advantage is greater than roughly the inverse of the effective population size (*N*_*e*_*s *> 1). In this case, the selective advantage to intron presence is related to the decreased mutation rate in the adjacent coding sequence. In the absence of recombination, the selective disadvantage to an allele that changes the mutation rate is roughly equal to the change in rate of mutation to disfavored alleles. So, if the general point mutation rate per generation is *u *and the difference in rate between intron-containing and intron-lacking alleles is *xu *per site, the selective advantage for having an intron which protects *l *adjacent sites, of which a fraction *c* is constrained by selection, will simply by *clxu*, and this selection will be sufficient to efficiently distinguish between intron-containing and intron-lacking alleles if *N*_*e*_*clxu *> 1.

Estimates of the product of the effective population size and the mutation rate (*N*_*e*_*u*) have been made for a range of eukaryotes, and vary from around 10^-2 ^to 10^-4 ^(most recently compiled by Lynch in MBE last year), thus we have the requirement *clx *> 10^2^-10^4^. For the lower value, this seems quite reasonable – if intron presence reduces the mutation rate by around twofold (i.e. *x *= 1) for *l *= 200 nucleotides of which around *c *= 0.5 are constrained, this would mean *clx *= 100, and all of these values could be quite conservative. Even values in the range of 10^4 ^seem quite not impossible: the condition would be fulfilled if a single intron protected *cl *= ~10,000 sites, or if *x *>> 1 (which may be more likely). Importantly, the hypothesis predicts that species with higher estimated *N*_*e*_*u *values should have more introns, directly opposite to the findings of Lynch (though as always correlations across available genomes are only as good as the genome sampling).

As such, I think that the hypothesis is viable overall and deserves to be widely read. I suspect that the manuscript's most important contribution will be in pointing the way for a new set of hypotheses based on newly positively selected traits of intron presence in early eukaryotes.

*Author response: I appreciate the comments from Dr. Roy. Frankly, my knowledge on intron and evolution is not enough to think the subject so deeply. This report is a very helpful enhancement of the section "Implications for intron evolution". I have not integrated this report in my manuscript as often done in revising manuscripts submitted to journals with anonymous review. The traditional reviewing model is unfair to anonymous reviewers even if the authors using some grateful words like, "as suggested by the anonymous reviewers, we...". I thank Biology Direct for providing such an efficient way for both authors and reviewers to contribute to the same subject, while both are indicated*.
